# Supplementary material for: Beyond the limits of the unassigned protist microbiome: inferring large-scale spatio-temporal patterns of Syndiniales marine parasites
Source: ISME Commun. 2023 Feb 28;3:16. doi: 10.1038/s43705-022-00203-7 (PMC9975217; doi:10.1038/s43705-022-00203-7)
Supplement: Supplementary file 1 — Supplementary information [file 43705_2022_203_MOESM1_ESM.pdf]

Supplementary Figures

Fig. S1: The 6 metabarcoding datasets gathered for this study. (A) Summary of homogenisation workflow and analysis for integrating the metabarcodes from the 6 metabarcoding datasets. Unassigned sequences kept after the homogenisation workflow were first studied through α-diversity measures within each dataset and then combined in the Sequence Similarity Network for spatiotemporal exploration (B, C) Maps indicating the sampling sites of each dataset. The number of different sampled locations for each dataset is: (B) ASTAN 1, BBMO 1, MOOSE 26, SOLA 1 ; (C) BioMarKs 6, Malaspina 122. The total number of different sampled locations in the global dataset is 155. (D) Timeline of the sampling periods covered by each metabarcoding datasets. The cumulative years of data in the global dataset covered by frequent sampling of the time-series correspond to 12 years of data. BioMarKs, Malaspina and MOOSE datasets include punctual samplings at the indicated years.

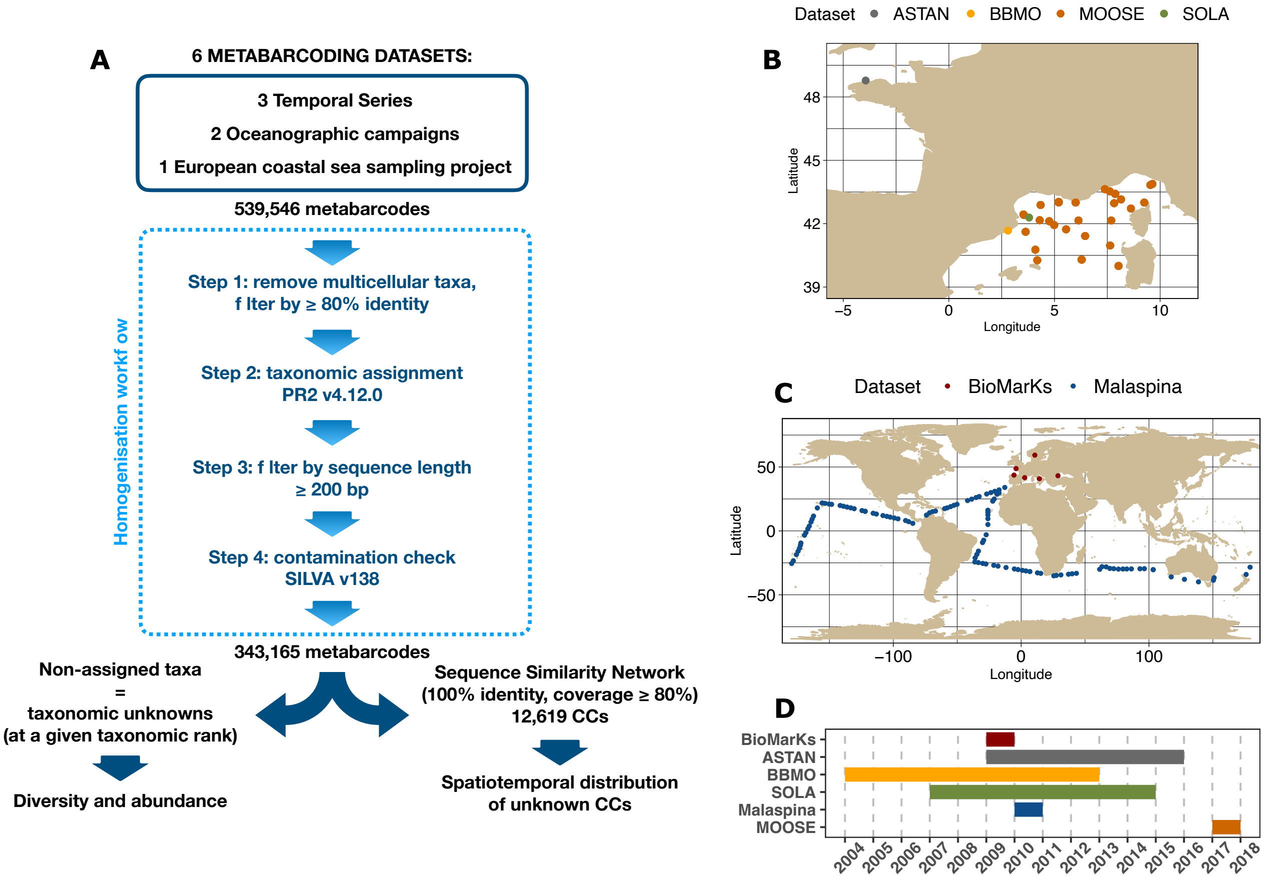

Fig. S2: Principal Component Analysis based on the metadata of the 6 studied metabarcoding datasets. PCA biplot with samples and physicochemical parameters (left): Chla (Chlorophyll α), O_2 (dissolved oxygen), Latitude, Longitude (spatial coordinates of the sampling spot), NO_2 (nitrate), NO_3 (nitrite), NH_4 (ammonium), PO_4 (phosphate), $SIOH_4$ (silicate), Depth, T (temperature), pH, S (salinity). The first 2 axes of the PCA explain 44.6% of metadata variation. PCA individuals (right) with samples colored by marine region. According to the first 2 axes of the PCA samples are grouped based on geographical location and depth, delimiting 6 marine regions.

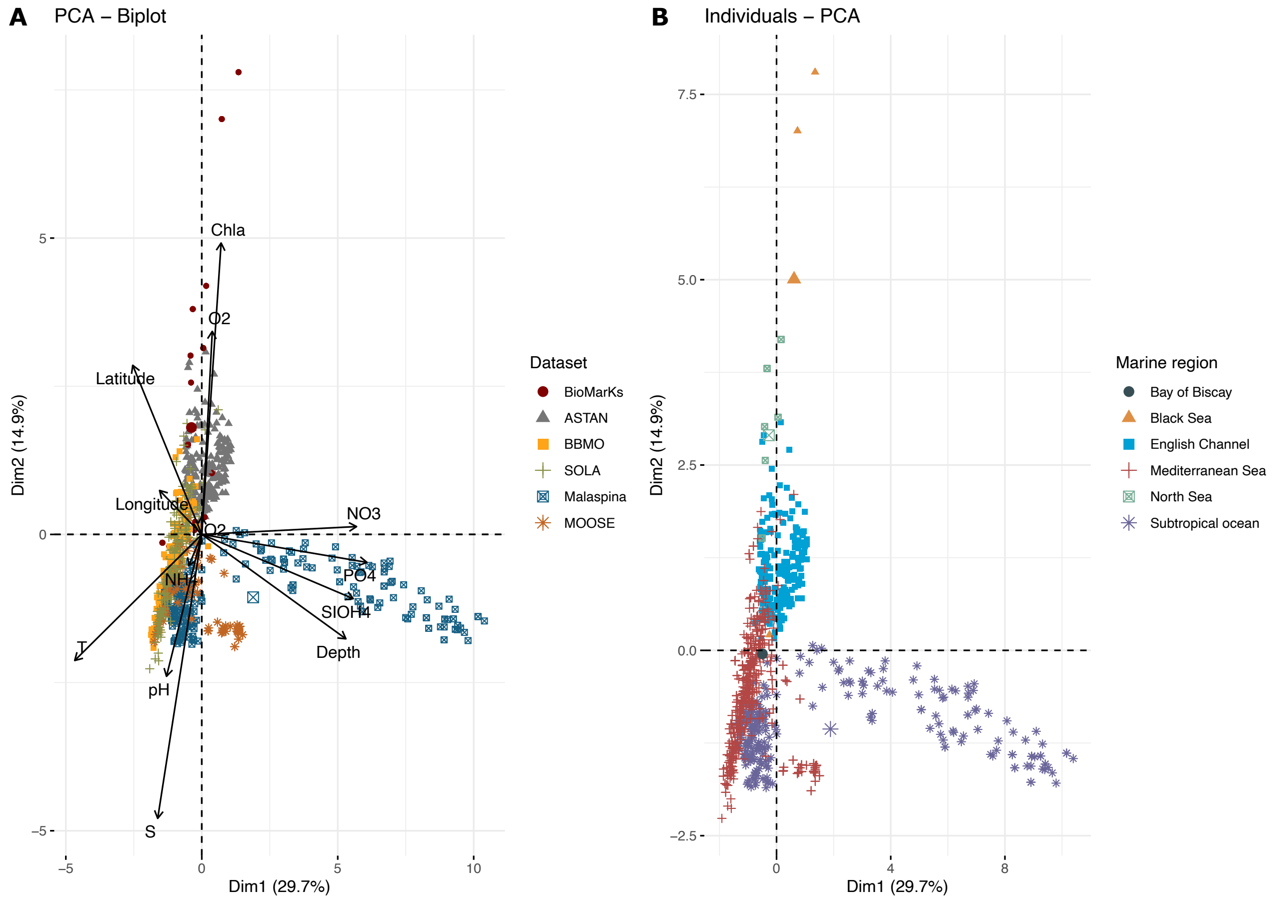

Fig. S3: Relative number of unassigned sequences across the taxonomic hierarchy (Kingdom to Species) for each dataset. Colors represent the 6 studied datasets. (A) Relative number of unassigned sequences across the homogenisation workflow: initial taxonomy of metabarcodes upon dataset receival (top); after taxonomic assignment with PR2 database v4.12.0 (middle; Fig. S1 (A) step 2); after filtering sequences by length and contamination check (bottom; Fig. S1 (A) step 3 & 4). (B) Relative number (top) and relative proportion (bottom) of unassigned sequences after the homogenisation workflow.

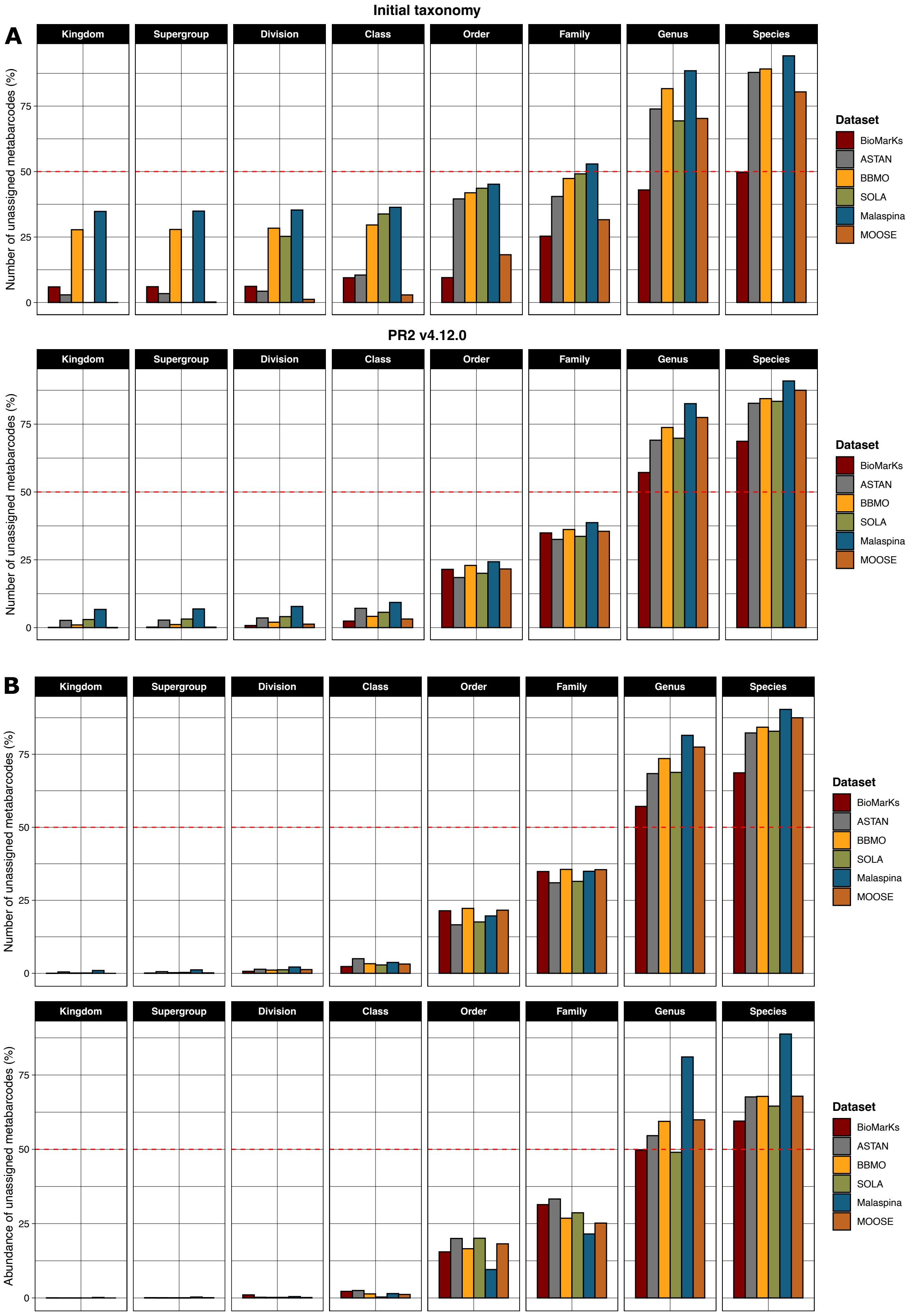

Fig. S4: Distribution of unassigned metabarcodes at kingdom level. (A) Map indicating the sampling sites of samples containing the studied metabarcodes. (B) Number of metabarcodes found across each dataset. 89.6% of metabarcodes are found in the Malaspina dataset. (C) Number of metabarcodes found across each depth type within the Malaspina dataset. The metabarcodes found in the bathypelagic layer represent 87.7% of metabarcodes unassigned at kingdom level.

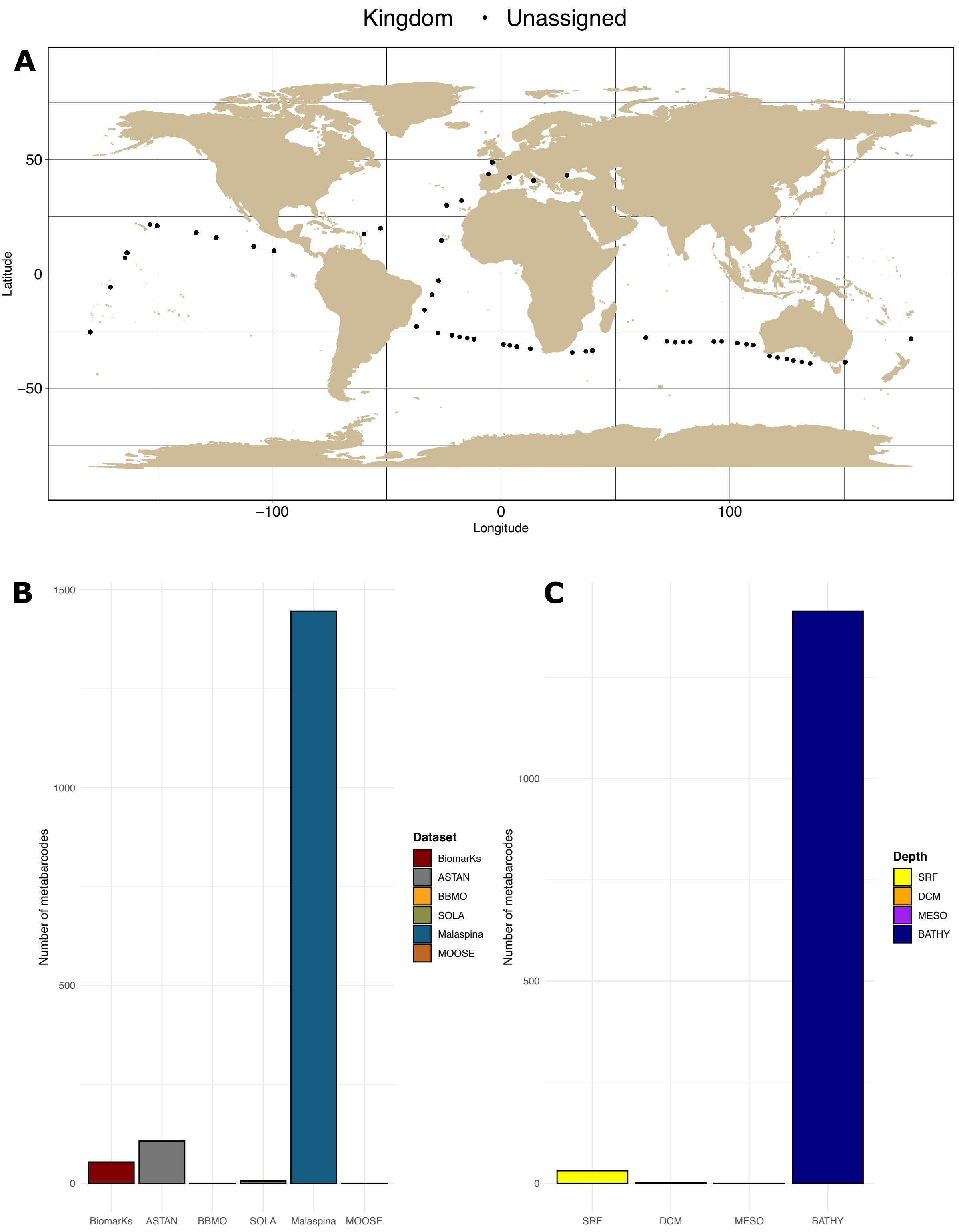

Fig. S5: A-diversity and abundance of metabarcodes lacking taxonomic annotation at the genus level, across datasets and major protist divisions. (A) Relative number (top) and relative abundance (bottom) of unassigned metabarcodes. Unassigned sequences are grouped according to their taxonomic annotation at the level of the Division (indicated by the color code). Only the top 4 most diverse / abundant divisions are plotted. The rest of divisions are included in “Others” and represent at no more than 6% of metabarcodes each. (B) Shannon diversity index of top protist divisions (in terms of diversity and abundance) for each dataset. The index was calculated taking into account both assigned and unassigned sequences (top), only assigned sequences (middle) and only unassigned sequences (bottom).

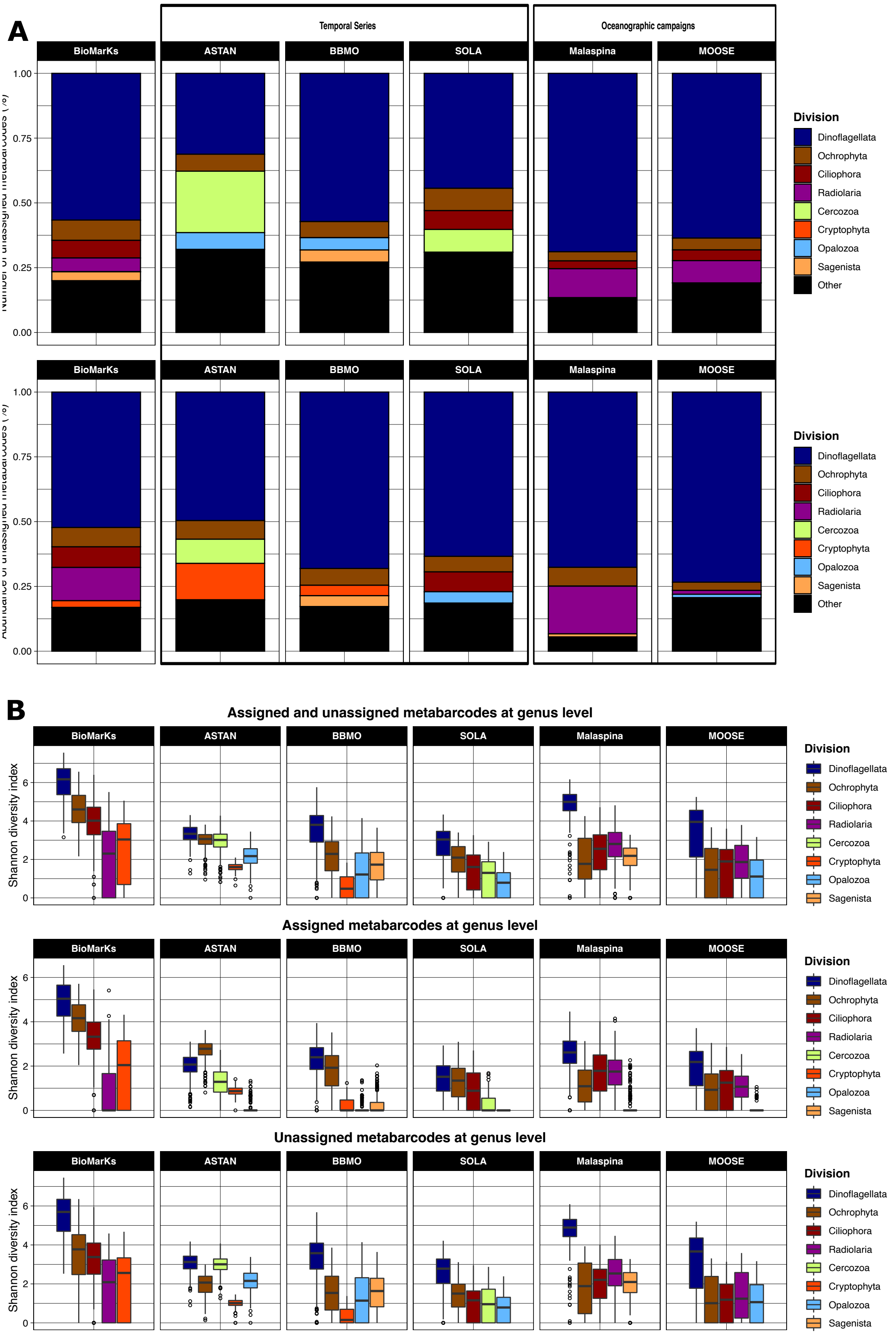

Fig. S6: Representativity of Dinoflagellata Classes in the 6 metabarcoding datasets. (A) Relative number (top) and relative abundance (bottom) of Dinoflagellata Classes. Others include Classes representing on average < 1% of Dinoflagellata metabarcodes in terms of number (n=3 655) and <1% in terms of abundance. These Classes are: Noctilucophyceae (3 287 metabarcodes), Oxyrrhea (1 metabarcode), Ellobiophyceae (12 metabarcodes) and sequences of Unknown class (no taxonomic assignment at Class level, 355 metabarcodes). (B) Relative number (top) and relative abundance (bottom) of assigned and unassigned sequences among Dinophyceae (left) and Syndiniales (right).

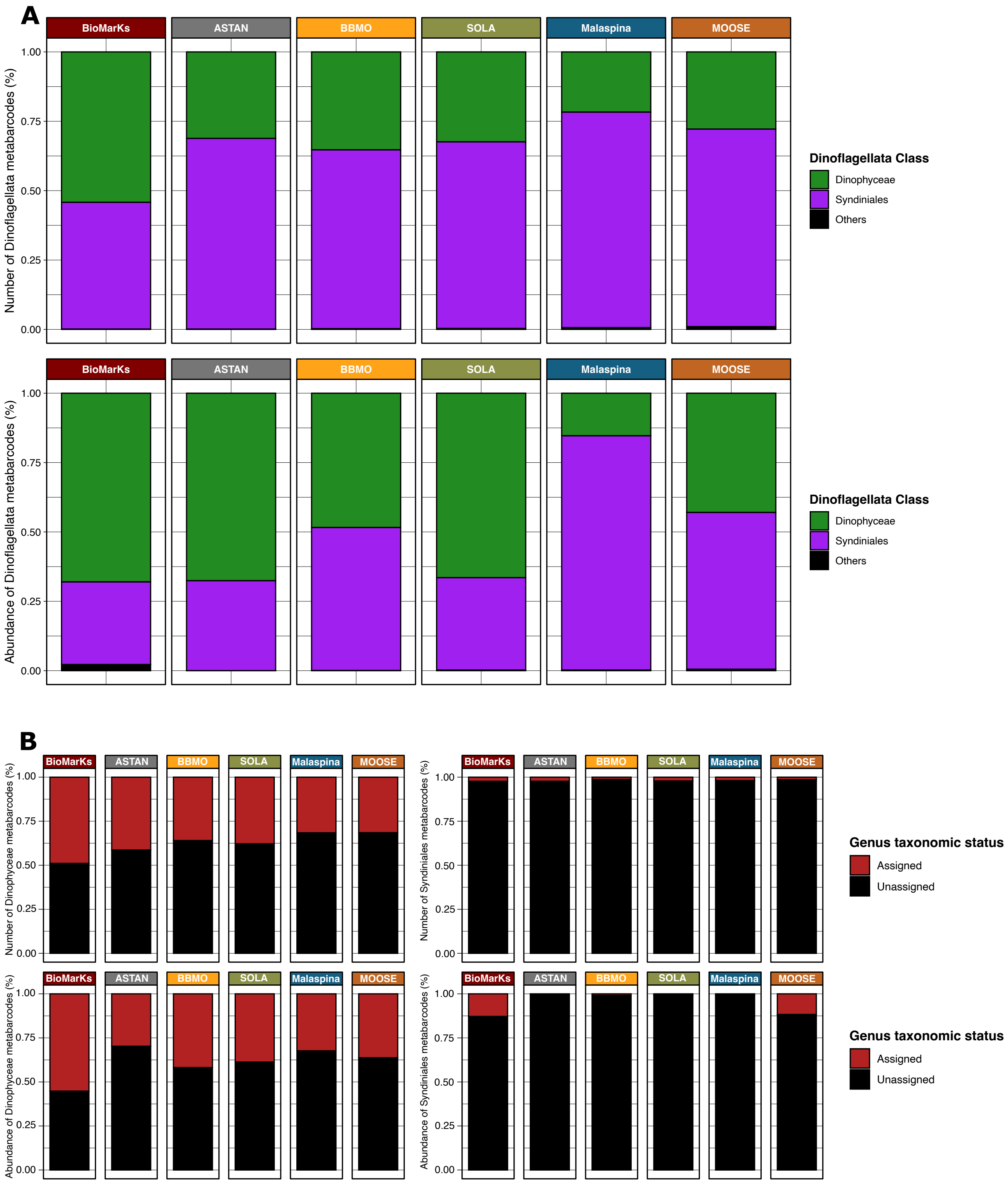

Fig. S7: Syndiniales CCs size and taxonomy. (A) Size distribution of Syndiniales according to their assignment at genus level: unassigned CCs (4 245 CCs, top) and assigned CCs (72 CCs, bottom). Scale bars differ between the two graphs. (B) Number of unassigned Syndiniales Connected Components (CCs) in the network (12 619 CCs) across Syndiniales orders. A CC is considered unassigned if it is composed of metabarcodes lacking taxonomic affiliation at the genus level. The network contained both assigned and unassigned CCs. The total number of Syndiniales CCs in the network is 4 317 (34.21% of the network) and the number of unassigned Syndiniales CCs is 4 245 (33.64% of the network; 47.60% of unassigned CCs of the network (i.e. 8 919 CCs); 98.33% of Syndiniales CCs). Syndiniales order IV (Dino-Group-IV) was composed of 41 CCs which contained only assigned sequences (genera *Hematodinium* and *Syndinium*) and was, thus, not plotted. "Unknown" corresponds to CCs composed of metabarcodes lacking taxonomic affiliation both at the order level.

A

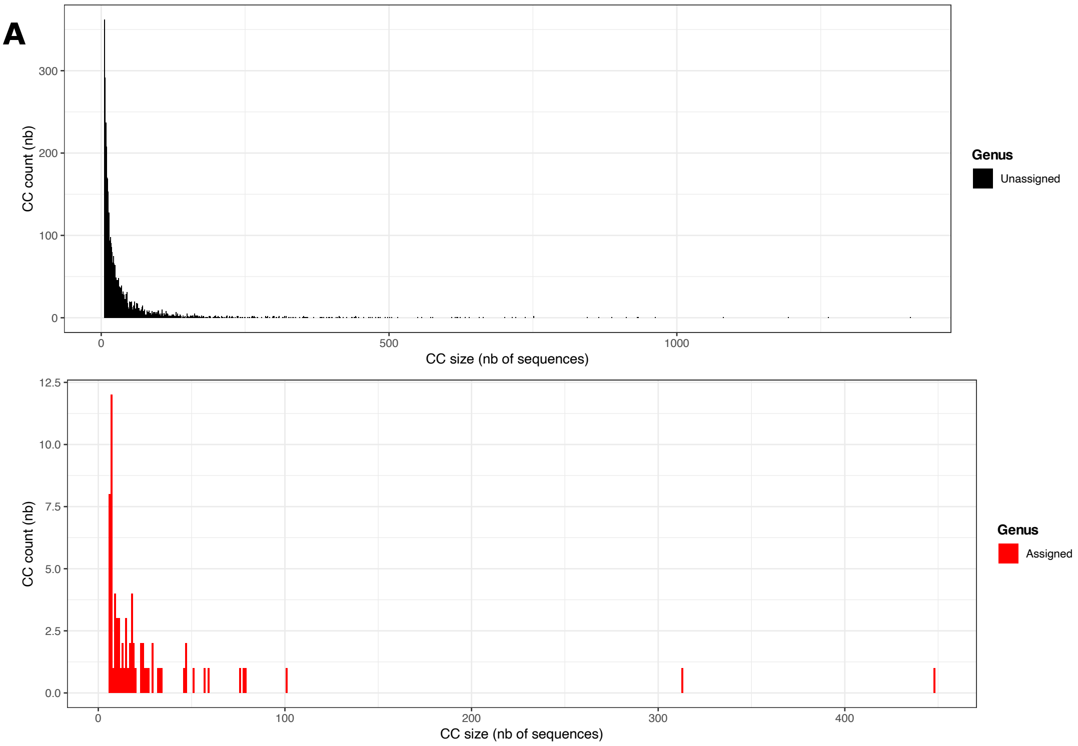

B

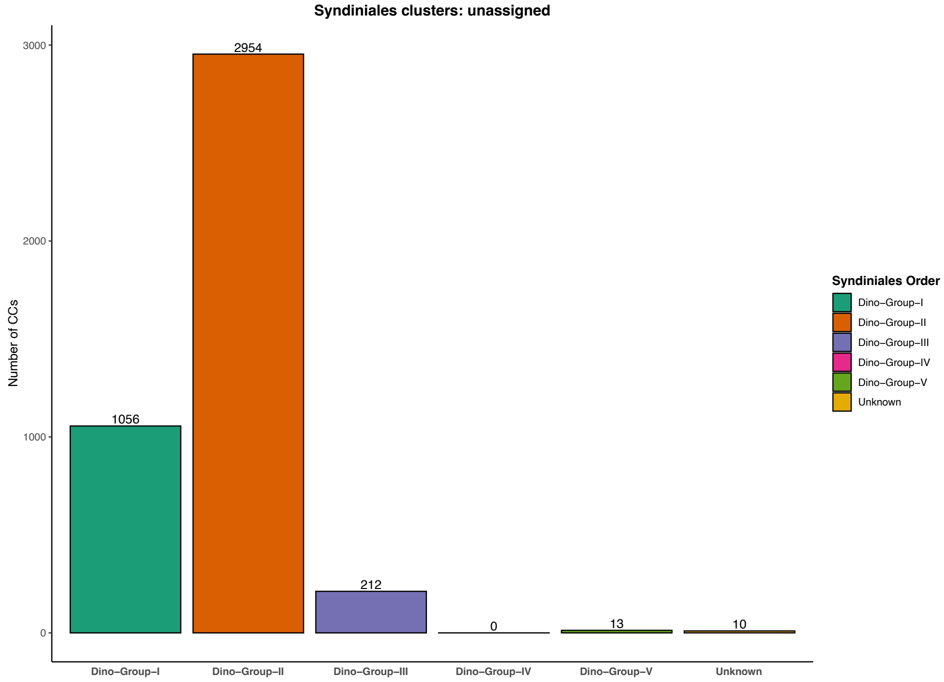

Fig. S8: Relative abundance of Syndiniales CCs across the 3 time-series: (A) ASTAN, (B) BBMO, (C) SOLA. The average monthly abundance is calculated per year and compiled as an average of the dataset. Colors indicate the presence/absence of taxonomic assignment of the CC at the genus level (red: presence of taxonomy; known genus and gray: absence of taxonomy; unassigned/unknown genus). Scale bars differ between the graphs. The big dots represent the CCs that were selected by the Escoufier's equivalent vectors and Lomb-Scargle periodogram algorithm methods for each time-series.

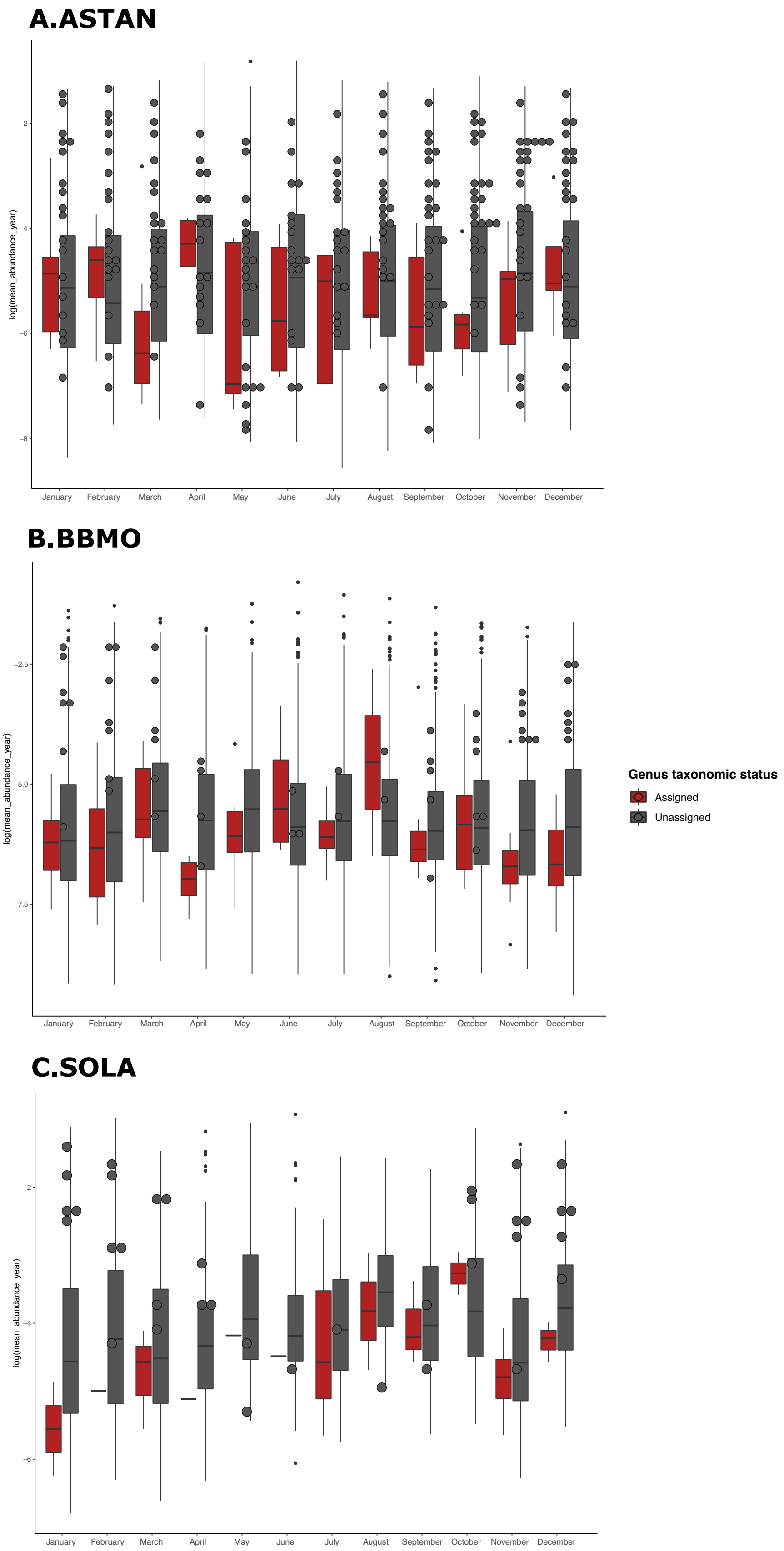

Supplementary Tables

Table S1: Information about the 6 metabarcoding datasets included in this study.

	BioMarKs	ASTAN	BBMO	SOLA	Malaspina	MOOSE	Global dataset
**Provider**	R Logares	M Caracciolo	R Logares	P Galand	R Logares	M Mendez-Sandin	-
**Abundance table**	Provided	Provided	Provided	Provided	Provided	Provided	-
**Taxonomic affiliation**	Provided	Provided	Provided	Provided	Provided	Provided	-
**DB version of starting taxonomy**	NA	PR2 v4.12.0	PR2 v4.11.1	SILVA v128	PR2 v4.11.1	PR2 v4.12.0	-
**Metadata origin**	SOMLIT, World Ocean Database, SeaDataNet	SOMLIT	Provided	Provided	Provided	Provided	-
**Samples (nb)**	115	374	327	154	289	272	1531
**Stations (nb)**	6	1	1	1	122	26	155
**Vertical profile max depth**	100	3	3	3	4000	2000	-
**Different months**	6	12	12	12	8	3	-
**Years**	1	8	10	8	1	2	-
**Sequencing**	454	Illumina MiSeq	Illumina MiSeq	Illumina MiSeq	Illumina MiSeq	Illumina MiSeq	-
**Clusters of reads**	OTU	ASV	ASV	ASV	ASV	ASV	-
**Read clustering threshold**	NA	NA	95 %	NA	95 %	80 %	-
**Applied read clustering threshold in this study**	80 %	80 %	-	80 %	-	80 %	-
**Starting number of metabarcodes**	423547	7933	59428	6400	59428	12279	569015
**Filtering step 1 (id > 80%, only protist sequences kept)**	239508	6239	29682	5554	59423	11322	351728
**Filtering step 2: taxonomic assignment specifications**	PR2 v4.12.0, eval 0.01	PR2 v4.12.0, eval 0.01	PR2 v4.12.0, eval 0.01	PR2 v4.12.0, eval 0.01	PR2 v4.12.0, eval 0.01	PR2 v4.12.0, eval 0.01	PR2 v4.12.0, eval 0.01
**Multicellular lineages removed**	Metazoa, Streptophyta, Florideophyceae, Bangiophyceae, Phaeophyceae, Ulvophyceae	Metazoa, Streptophyta, Florideophyceae, Bangiophyceae, Phaeophyceae, Ulvophyceae	Metazoa, Streptophyta, Florideophyceae, Bangiophyceae, Phaeophyceae, Ulvophyceae	Metazoa, Streptophyta, Florideophyceae, Bangiophyceae, Phaeophyceae, Ulvophyceae	Metazoa, Streptophyta, Florideophyceae, Bangiophyceae, Phaeophyceae, Ulvophyceae	Metazoa, Streptophyta, Florideophyceae, Bangiophyceae, Phaeophyceae, Ulvophyceae	Metazoa, Streptophyta, Florideophyceae, Bangiophyceae, Phaeophyceae, Ulvophyceae
**Filtering step 3 (sequence length > 200 bp)**	236618	6213	29073	5276	58555	11304	347039
**Filtering step 4 (contamination check with SILVA v138)**	236575	6087	28809	5218	55173	11303	343165

Table S2: Jaccard diversity index calculated between sea regions for the following protist groups: Dinophyeae, Ciliophora, Radiolaria, Ochrophyta, Cercozoa, Cryptophyta, Opalozoa, Sagenista and Syndiniales.

Dinophyceae	Bay.of.Biscay	Black.Sea	English.Channel	Mediterranean.Sea	North.Sea	Subtropical.ocean
Bay of Biscay	0	0.938173454130847	0.87497104538359	0.804672833794989	0.927670261180023	0.818861390415376
Black Sea	0.938173454130847	0	0.901578438140029	0.890869330127625	0.791834766178996	0.962644200953342
English Channel	0.87497104538359	0.901578438140029	0	0.798497443157509	0.859471772489166	0.894607381909587
Mediterranean Sea	0.804672833794989	0.890869330127625	0.798497443157509	0	0.867745884222841	0.664604534857357
North Sea	0.927670261180023	0.791834766178996	0.859471772489166	0.867745884222841	0	0.954859723071374
Subtropical ocean	0.818861390415376	0.962644200953342	0.894607381909587	0.664604534857357	0.954859723071374	0
**Ciliophora**	Bay.of.Biscay	Black.Sea	English.Channel	Mediterranean.Sea	North.Sea	Subtropical.ocean
Bay of Biscay	0	0.867381892806053	0.902947145619635	0.842217097011825	0.872886640938259	0.858727477402051
Black Sea	0.867381892806053	0	0.887898854315709	0.891501486515274	0.79049701022653	0.944252209613938
English Channel	0.902947145619635	0.887898854315709	0	0.777126046175384	0.803651192794572	0.923737512275255
Mediterranean Sea	0.842217097011825	0.891501486515274	0.777126046175384	0	0.828880954082161	0.729483694176171
North Sea	0.872886640938259	0.79049701022653	0.803651192794572	0.828880954082161	0	0.92877157288191
Subtropical ocean	0.858727477402051	0.944252209613938	0.923737512275255	0.729483694176171	0.92877157288191	0
**Radiolaria**	Bay.of.Biscay	Black.Sea	English.Channel	Mediterranean.Sea	North.Sea	Subtropical.ocean
Bay of Biscay	0	0.97462797037596	0.870508797970726	0.942580875095232	0.914028700372153	0.98111749754938
Black Sea	0.97462797037596	0	0.958390416328276	0.990831276360653	0.983866226229662	0.991593478316324
English Channel	0.870508797970726	0.958390416328276	0	0.961150318767074	0.899296029993727	0.986294136252654
Mediterranean Sea	0.942580875095232	0.990831276360653	0.961150318767074	0	0.896220133271001	0.727417292423973
North Sea	0.914028700372153	0.983866226229662	0.899296029993727	0.896220133271001	0	0.993423735613338
Subtropical ocean	0.98111749754938	0.991593478316324	0.986294136252654	0.727417292423973	0.993423735613338	0
**Ochrophyta**	Bay.of.Biscay	Black.Sea	English.Channel	Mediterranean.Sea	North.Sea	Subtropical.ocean
Bay of Biscay	0	0.944213049494936	0.953554574430131	0.862135906227205	0.939353323258805	0.839757547138781
Black Sea	0.944213049494936	0	0.918409472383869	0.892072704102568	0.732692288501858	0.961403091261643
English Channel	0.953554574430131	0.918409472383869	0	0.806755268471589	0.882128402710598	0.953440648813368
Mediterranean Sea	0.862135906227205	0.892072704102568	0.806755268471589	0	0.83361488993896	0.834034881022253
North Sea	0.939353323258805	0.732692288501858	0.882128402710598	0.83361488993896	0	0.965028633968432
Subtropical ocean	0.839757547138781	0.961403091261643	0.953440648813368	0.834034881022253	0.965028633968432	0
**Cercozoa**	Bay.of.Biscay	Black.Sea	English.Channel	Mediterranean.Sea	North.Sea	Subtropical.ocean
Bay of Biscay	0	0.982103174330624	0.96701345562615	0.878828971450338	0.904605092480974	0.930785824223574
Black Sea	0.982103174330624	0	0.951501456544751	0.927806049444131	0.877524702758534	0.99158348108881
English Channel	0.96701345562615	0.951501456544751	0	0.745475896500063	0.843165738440751	0.967596506393834
Mediterranean Sea	0.878828971450338	0.927806049444131	0.745475896500063	0	0.837231980758612	0.88421222368964
North Sea	0.904605092480974	0.877524702758534	0.843165738440751	0.837231980758612	0	0.981476463546255
Subtropical ocean	0.930785824223574	0.99158348108881	0.967596506393834	0.88421222368964	0.981476463546255	0
**Cryptophyta**	Bay.of.Biscay	Black.Sea	English.Channel	Mediterranean.Sea	North.Sea	Subtropical.ocean
Bay of Biscay	0	0.852266116053246	0.881612974786431	0.747806749032386	0.944704795765131	0.791666798740924
Black Sea	0.852266116053246	0	0.821118584548414	0.735923947815264	0.671563329597875	0.928054816498048
English Channel	0.881612974786431	0.821118584548414	0	0.786600033491997	0.813523218679703	0.870377288638509
Mediterranean Sea	0.747806749032386	0.735923947815264	0.786600033491997	0	0.850928644235467	0.882593230983105
North Sea	0.944704795765131	0.671563329597875	0.813523218679703	0.850928644235467	0	0.938662701091859
Subtropical ocean	0.791666798740924	0.928054816498048	0.870377288638509	0.882593230983105	0.938662701091859	0
**Opalozoa**	Bay.of.Biscay	Black.Sea	English.Channel	Mediterranean.Sea	North.Sea	Subtropical.ocean
Bay of Biscay	0	1	0.900792047825135	0.810741256648265	0.915114872445857	0.904576802623282
Black Sea	1	0	0.924648613090138	0.976234364064496	0.874080566513386	0.971848215239348
English Channel	0.900792047825135	0.924648613090138	0	0.745279921844327	0.770950207818785	0.90305636237645
Mediterranean Sea	0.810741256648265	0.976234364064496	0.745279921844327	0	0.839940179196507	0.843786084383779

**North Sea**	0.915114872445857	0.874080566513386	0.770950207818785	0.839940179196507	0	0.957003482122313
**Subtropical ocean**	0.904576802623282	0.971848215239348	0.90305636237645	0.843786084383779	0.957003482122313	0
**Sagenista**	Bay.of.Biscay	Black.Sea	English.Channel	Mediterranean.Sea	North.Sea	Subtropical.ocean
**Bay of Biscay**	0	0.927494765810393	0.812106830102491	0.746219107052659	0.847799688731939	0.760141643941602
**Black Sea**	0.927494765810393	0	0.83502956899843	0.927283159174829	0.704648885459417	0.970443781190252
**English Channel**	0.812106830102491	0.83502956899843	0	0.738032807799124	0.682014630383829	0.845932127615074
**Mediterranean Sea**	0.746219107052659	0.927283159174829	0.738032807799124	0	0.809437286678021	0.620999021505498
**North Sea**	0.847799688731939	0.704648885459417	0.682014630383829	0.809437286678021	0	0.904155003240767
**Subtropical ocean**	0.760141643941602	0.970443781190252	0.845932127615074	0.620999021505498	0.904155003240767	0
**Syndiniales**	Bay.of.Biscay	Black.Sea	English.Channel	Mediterranean.Sea	North.Sea	Subtropical.ocean
**Bay of Biscay**	0	0.940557162491321	0.872991324662993	0.867376931604074	0.910315399979933	0.898494917470853
**Black Sea**	0.940557162491321	0	0.91577064018812	0.949739577024946	0.817131780391512	0.96862939955675
**English Channel**	0.872991324662993	0.91577064018812	0	0.814610404707088	0.84839192562793	0.872386318763916
**Mediterranean Sea**	0.867376931604074	0.949739577024946	0.814610404707088	0	0.902815004053065	0.60089678886582
**North Sea**	0.910315399979933	0.817131780391512	0.84839192562793	0.902815004053065	0	0.935820962510432
**Subtropical ocean**	0.898494917470853	0.96862939955675	0.872386318763916	0.60089678886582	0.935820962510432	0

Table S3: Indicator CCs indicated by the Escouffier's equivalent method.

	Dataset	CC_id	Escouffier	RV
**1**	ASTAN	CC_unknown_535	1	0.248
**2**	ASTAN	CC_unknown_154	2	0.32
**3**	ASTAN	CC_unknown_532	3	0.372
**4**	ASTAN	CC_unknown_168	4	0.409
**5**	ASTAN	CC_unknown_547	5	0.442
**6**	ASTAN	CC_unknown_368	6	0.471
**7**	ASTAN	CC_unknown_13	7	0.491
**8**	ASTAN	CC_unknown_109	8	0.511
**9**	ASTAN	CC_unknown_363	9	0.53
**10**	ASTAN	CC_unknown_257	10	0.544
**11**	ASTAN	CC_unknown_498	11	0.557
**12**	ASTAN	CC_unknown_370	12	0.57
**13**	ASTAN	CC_unknown_244	13	0.581
**14**	ASTAN	CC_unknown_172	14	0.592
**15**	ASTAN	CC_unknown_77	15	0.602
**16**	ASTAN	CC_unknown_210	16	0.611
**17**	ASTAN	CC_known_12	17	0.619
**18**	ASTAN	CC_unknown_183	18	0.627
**19**	ASTAN	CC_unknown_550	19	0.634
**20**	ASTAN	CC_unknown_35	20	0.641
**21**	ASTAN	CC_unknown_30	21	0.648
**22**	ASTAN	CC_unknown_254	22	0.655
**23**	ASTAN	CC_unknown_80	23	0.661
**24**	ASTAN	CC_unknown_307	24	0.667
**25**	ASTAN	CC_unknown_441	25	0.672
**26**	ASTAN	CC_unknown_462	26	0.678
**27**	ASTAN	CC_unknown_530	27	0.683
**28**	ASTAN	CC_unknown_126	28	0.689
**29**	ASTAN	CC_unknown_328	29	0.693
**30**	ASTAN	CC_unknown_209	30	0.697
**31**	ASTAN	CC_unknown_134	31	0.702
**32**	ASTAN	CC_unknown_339	32	0.706
**33**	ASTAN	CC_unknown_272	33	0.71
**34**	ASTAN	CC_unknown_496	34	0.714
**35**	ASTAN	CC_unknown_295	35	0.717
**36**	ASTAN	CC_unknown_483	36	0.721
**37**	ASTAN	CC_unknown_553	37	0.725
**38**	ASTAN	CC_unknown_20	38	0.728
**39**	ASTAN	CC_unknown_12	39	0.731
**40**	ASTAN	CC_unknown_349	40	0.734
**41**	ASTAN	CC_unknown_58	41	0.737
**42**	ASTAN	CC_unknown_62	42	0.74
**43**	ASTAN	CC_unknown_362	43	0.743
**44**	ASTAN	CC_unknown_227	44	0.746
**45**	ASTAN	CC_unknown_320	45	0.749
**46**	BBMO	CC_unknown_72	1	0.261
**47**	BBMO	CC_unknown_330	2	0.344
**48**	BBMO	CC_unknown_112	3	0.403
**49**	BBMO	CC_unknown_562	4	0.442
**50**	BBMO	CC_unknown_213	5	0.478
**51**	BBMO	CC_unknown_2182	6	0.507
**52**	BBMO	CC_unknown_1964	7	0.532
**53**	BBMO	CC_unknown_401	8	0.552
**54**	BBMO	CC_unknown_1225	9	0.567
**55**	BBMO	CC_unknown_926	10	0.58
**56**	BBMO	CC_unknown_129	11	0.592
**57**	BBMO	CC_unknown_389	12	0.604
**58**	BBMO	CC_unknown_154	13	0.615
**59**	BBMO	CC_unknown_767	14	0.626
**60**	BBMO	CC_unknown_1451	15	0.636
**61**	BBMO	CC_unknown_1869	16	0.645
**62**	BBMO	CC_unknown_1827	17	0.653
**63**	BBMO	CC_unknown_1514	18	0.661
**64**	BBMO	CC_unknown_1868	19	0.668
**65**	BBMO	CC_unknown_1039	20	0.675
**66**	BBMO	CC_unknown_1532	21	0.68

**67**	BBMO	CC_unknown_482	22	0.686
**68**	BBMO	CC_unknown_2139	23	0.691
**69**	BBMO	CC_unknown_404	24	0.696
**70**	BBMO	CC_unknown_989	25	0.701
**71**	BBMO	CC_unknown_604	26	0.706
**72**	BBMO	CC_unknown_161	27	0.711
**73**	BBMO	CC_unknown_1960	28	0.716
**74**	BBMO	CC_unknown_2212	29	0.721
**75**	BBMO	CC_unknown_839	30	0.725
**76**	BBMO	CC_unknown_894	31	0.729
**77**	BBMO	CC_unknown_1775	32	0.733
**78**	BBMO	CC_unknown_1893	33	0.737
**79**	BBMO	CC_unknown_296	34	0.74
**80**	BBMO	CC_unknown_1563	35	0.744
**81**	BBMO	CC_unknown_126	36	0.747
**82**	SOLA	CC_unknown_425	1	0.341
**83**	SOLA	CC_unknown_164	2	0.436
**84**	SOLA	CC_unknown_918	3	0.512
**85**	SOLA	CC_unknown_126	4	0.551
**86**	SOLA	CC_unknown_199	5	0.579
**87**	SOLA	CC_unknown_149	6	0.604
**88**	SOLA	CC_unknown_326	7	0.625
**89**	SOLA	CC_unknown_39	8	0.643
**90**	SOLA	CC_unknown_892	9	0.658
**91**	SOLA	CC_unknown_171	10	0.673
**92**	SOLA	CC_unknown_226	11	0.686
**93**	SOLA	CC_unknown_1793	12	0.697
**94**	SOLA	CC_unknown_143	13	0.707
**95**	SOLA	CC_unknown_183	14	0.717
**96**	SOLA	CC_unknown_333	15	0.728
**97**	SOLA	CC_unknown_293	16	0.737
**98**	SOLA	CC_unknown_1908	17	0.747

Table S4: Rhythmic CCs indicated by the Lomb-Scargle Periodogram (LSP) method.

	Dataset	CC_id	PNmax	Pvalue	Period
1	ASTAN	CC_known_1	14.99	1E-04	363.25
2	ASTAN	CC_known_14	38.403	0	372.56
3	ASTAN	CC_known_2	13.533	5E-04	854.71
4	ASTAN	CC_known_4	10.914	0.0068	372.56
5	ASTAN	CC_unknown_1	15.19	1E-04	392.7
6	ASTAN	CC_unknown_10	16.506	0	372.56
7	ASTAN	CC_unknown_100	28.65	0	372.56
8	ASTAN	CC_unknown_103	34.521	0	372.56
9	ASTAN	CC_unknown_104	11.247	0.0049	363.25
10	ASTAN	CC_unknown_106	15.962	0	363.25
11	ASTAN	CC_unknown_107	10.778	0.0078	363.25
12	ASTAN	CC_unknown_11	27.452	0	363.25
13	ASTAN	CC_unknown_110	10.851	0.0072	454.06
14	ASTAN	CC_unknown_111	11.814	0.0028	2906
15	ASTAN	CC_unknown_112	11.592	0.0034	345.95
16	ASTAN	CC_unknown_113	10.096	0.0152	363.25
17	ASTAN	CC_unknown_116	13.206	7E-04	2421.7
18	ASTAN	CC_unknown_118	14.369	2E-04	363.25
19	ASTAN	CC_unknown_119	59.04	0	372.56
20	ASTAN	CC_unknown_12	13.212	7E-04	354.39
21	ASTAN	CC_unknown_120	16.056	0	726.5
22	ASTAN	CC_unknown_121	10.003	0.0167	363.25
23	ASTAN	CC_unknown_122	10.615	0.0091	345.95
24	ASTAN	CC_unknown_123	10.134	0.0147	220.15
25	ASTAN	CC_unknown_125	11.599	0.0034	372.56
26	ASTAN	CC_unknown_126	30.339	0	2906
27	ASTAN	CC_unknown_128	38.644	0	363.25
28	ASTAN	CC_unknown_129	18.939	0	372.56
29	ASTAN	CC_unknown_13	102.62	0	363.25
30	ASTAN	CC_unknown_131	10.42	0.011	345.95
31	ASTAN	CC_unknown_132	32.41	0	363.25
32	ASTAN	CC_unknown_134	47.33	0	363.25

33	ASTAN	CC_unknown_135	14.175	3E-04	372.56
34	ASTAN	CC_unknown_138	20.178	0	354.39
35	ASTAN	CC_unknown_14	13.808	4E-04	363.25
36	ASTAN	CC_unknown_141	11.711	0.0031	363.25
37	ASTAN	CC_unknown_142	18.257	0	183.92
38	ASTAN	CC_unknown_143	57.76	0	372.56
39	ASTAN	CC_unknown_145	13.188	7E-04	363.25
40	ASTAN	CC_unknown_146	12.62	0.0012	354.39
41	ASTAN	CC_unknown_147	17.288	0	372.56
42	ASTAN	CC_unknown_149	48.702	0	372.56
43	ASTAN	CC_unknown_15	17.183	0	372.56
44	ASTAN	CC_unknown_151	10.445	0.0108	363.25
45	ASTAN	CC_unknown_153	15.689	1E-04	372.56
46	ASTAN	CC_unknown_154	41.522	0	363.25
47	ASTAN	CC_unknown_155	18.615	0	372.56
48	ASTAN	CC_unknown_158	21.839	0	363.25
49	ASTAN	CC_unknown_16	10.074	0.0156	1816.2
50	ASTAN	CC_unknown_164	22.278	0	372.56
51	ASTAN	CC_unknown_167	39.31	0	372.56
52	ASTAN	CC_unknown_168	10.56	0.0097	392.7
53	ASTAN	CC_unknown_169	10.356	0.0118	372.56
54	ASTAN	CC_unknown_17	52.237	0	372.56
55	ASTAN	CC_unknown_170	57.889	0	363.25
56	ASTAN	CC_unknown_172	33.36	0	181.62
57	ASTAN	CC_unknown_174	10.436	0.0109	354.39
58	ASTAN	CC_unknown_181	18.714	0	372.56
59	ASTAN	CC_unknown_182	11.327	0.0045	363.25
60	ASTAN	CC_unknown_183	63.614	0	363.25
61	ASTAN	CC_unknown_185	12.843	0.001	372.56
62	ASTAN	CC_unknown_186	10.663	0.0087	363.25
63	ASTAN	CC_unknown_189	73.299	0	363.25
64	ASTAN	CC_unknown_19	10.776	0.0078	363.25
65	ASTAN	CC_unknown_191	58.068	0	363.25
66	ASTAN	CC_unknown_194	13.648	4E-04	363.25

**67**	ASTAN	CC_unknown_199	84.242	0	363.25
**68**	ASTAN	CC_unknown_2	21.007	0	2421.7
**69**	ASTAN	CC_unknown_20	34.274	0	363.25
**70**	ASTAN	CC_unknown_200	11.665	0.0032	363.25
**71**	ASTAN	CC_unknown_203	67.627	0	363.25
**72**	ASTAN	CC_unknown_204	10.212	0.0136	354.39
**73**	ASTAN	CC_unknown_205	10.007	0.0167	354.39
**74**	ASTAN	CC_unknown_21	11.216	0.005	363.25
**75**	ASTAN	CC_unknown_215	10.581	0.0095	372.56
**76**	ASTAN	CC_unknown_22	12.19	0.0019	372.56
**77**	ASTAN	CC_unknown_227	13.81	4E-04	363.25
**78**	ASTAN	CC_unknown_229	10.328	0.0121	363.25
**79**	ASTAN	CC_unknown_231	12.65	0.0012	354.39
**80**	ASTAN	CC_unknown_24	17.843	0	372.56
**81**	ASTAN	CC_unknown_243	13.767	4E-04	2421.7
**82**	ASTAN	CC_unknown_250	49.844	0	363.25
**83**	ASTAN	CC_unknown_255	12.458	0.0014	181.62
**84**	ASTAN	CC_unknown_257	77.824	0	363.25
**85**	ASTAN	CC_unknown_26	39.7	0	354.39
**86**	ASTAN	CC_unknown_260	13.466	5E-04	363.25
**87**	ASTAN	CC_unknown_264	43.049	0	372.56
**88**	ASTAN	CC_unknown_27	10.072	0.0156	16.06
**89**	ASTAN	CC_unknown_272	73.939	0	372.56
**90**	ASTAN	CC_unknown_28	12.63	0.0012	363.25
**91**	ASTAN	CC_unknown_283	15.732	1E-04	2906
**92**	ASTAN	CC_unknown_299	10.271	0.0128	363.25
**93**	ASTAN	CC_unknown_3	17.043	0	363.25
**94**	ASTAN	CC_unknown_30	17.471	0	1614.4
**95**	ASTAN	CC_unknown_300	55.067	0	363.25
**96**	ASTAN	CC_unknown_306	13.967	3E-04	354.39
**97**	ASTAN	CC_unknown_31	12.441	0.0015	726.5
**98**	ASTAN	CC_unknown_313	25.045	0	363.25
**99**	ASTAN	CC_unknown_32	11.778	0.0029	363.25
**10(**	ASTAN	CC_unknown_325	45.552	0	354.39

Table S5: List of CCs selected both by Escouffier's equivalent vectors and LSP methods.

	Dataset	CC_id	Escouffier	RV	PNmax	Pvalue	Period	Periodicity
**1**	ASTAN	CC_unknown_535	1	0.248	99.725	0	372.56	1
**2**	ASTAN	CC_unknown_154	2	0.32	41.522	0	363.25	1
**3**	ASTAN	CC_unknown_532	3	0.372	89.553	0	363.25	1
**4**	ASTAN	CC_unknown_168	4	0.409	10.56	0.0097	392.7	1
**5**	ASTAN	CC_unknown_547	5	0.442	13.203	7E-04	354.39	1
**7**	ASTAN	CC_unknown_13	7	0.491	102.62	0	363.25	1
**10**	ASTAN	CC_unknown_257	10	0.544	77.824	0	363.25	1
**11**	ASTAN	CC_unknown_498	11	0.557	33.229	0	363.25	1
**12**	ASTAN	CC_unknown_370	12	0.57	40.716	0	363.25	1
**14**	ASTAN	CC_unknown_172	14	0.592	33.36	0	181.62	1
**18**	ASTAN	CC_unknown_183	18	0.627	63.614	0	363.25	1
**19**	ASTAN	CC_unknown_550	19	0.634	45.499	0	363.25	1
**20**	ASTAN	CC_unknown_35	20	0.641	11.1	0.0056	2906	1
**21**	ASTAN	CC_unknown_30	21	0.648	17.471	0	1614.4	1
**23**	ASTAN	CC_unknown_80	23	0.661	16.021	0	363.25	1
**26**	ASTAN	CC_unknown_462	26	0.678	19.424	0	354.39	1
**27**	ASTAN	CC_unknown_530	27	0.683	32.635	0	354.39	1
**28**	ASTAN	CC_unknown_126	28	0.689	30.339	0	2906	1
**31**	ASTAN	CC_unknown_134	31	0.702	47.33	0	363.25	1
**33**	ASTAN	CC_unknown_272	33	0.71	73.939	0	372.56	1
**36**	ASTAN	CC_unknown_483	36	0.721	39.84	0	354.39	1
**37**	ASTAN	CC_unknown_553	37	0.725	10.356	0.0118	16.06	1
**38**	ASTAN	CC_unknown_20	38	0.728	34.274	0	363.25	1
**39**	ASTAN	CC_unknown_12	39	0.731	13.212	7E-04	354.39	1
**41**	ASTAN	CC_unknown_58	41	0.737	15.912	0	354.39	1
**42**	ASTAN	CC_unknown_62	42	0.74	30.798	0	807.22	1
**44**	ASTAN	CC_unknown_227	44	0.746	13.81	4E-04	363.25	1
**48**	BBMO	CC_unknown_112	3	0.403	16.829	0	515.86	1
**50**	BBMO	CC_unknown_213	5	0.478	11.852	0.0023	487.97	1
**53**	BBMO	CC_unknown_401	8	0.552	14.165	2E-04	501.53	1
**57**	BBMO	CC_unknown_389	12	0.604	10.489	0.0089	501.53	1
**58**	BBMO	CC_unknown_154	13	0.615	15.424	1E-04	515.86	1

**79**	BBMO	CC_unknown_296	34	0.74	20.699	0	501.53	1
**81**	BBMO	CC_unknown_126	36	0.747	13.3	5E-04	515.86	1
**82**	SOLA	CC_unknown_425	1	0.341	16.862	0	360.5	1
**85**	SOLA	CC_unknown_126	4	0.551	23.175	0	360.5	1
**87**	SOLA	CC_unknown_149	6	0.604	14.401	1E-04	372.93	1
**94**	SOLA	CC_unknown_143	13	0.707	12.967	3E-04	360.5	1
**95**	SOLA	CC_unknown_183	14	0.717	16.434	0	360.5	1

Table S6: Summary information of temporal analysis.

	ASTAN	BBMO	SOLA	Sum
**Nb Syndiniales CCs**	572	2171	566	3309
**Nb unassigned Syndiniales CCs**	558	2142	556	3256
**Nb CCs selected by Escoufier vectors**	45	36	17	98
**Nb CCs selected by Lomb-Scargle algorithm**	208	118	15	341
**Average recurrence period (days)**	570	578	365	-
**Min recurrence period (days)**	16	23	360	-
**Max recurrence period (days)**	2906	3611	372	-
**Nb CCs selected by Escoufiers vectors and Lomb-Scargle algorithm***	27	7	5	39
**Nb selected* CCs present in the three time-series**	1	1	1	-
**Selected* CCs ids present in the three time-series**	CC_unknown_126	CC_unknown_126	CC_unknown_126	-
**Recurrence period (days)**	2906	515	360	-
**Nb selected* CCs present in ASTAN and BBMO**	1	1	-	-
**Selected* CCs ids present in ASTAN and BBMO**	CC_unknown_154	CC_unknown_154	-	-
**Recurrence period (days)**	363	515	-	-
**Nb selected* CCs present in ASTAN and SOLA**	1	-	1	-
**Selected* CCs ids present in ASTAN and SOLA**	CC_unknown_183	-	CC_unknown_183	-
**Recurrence period (days)**	360	-	363	-
**Nb selected* CCs present in BBMO and SOLA**	-	0	0	-
**Selected* CCs ids present in BBMO and SOLA**	-	-	-	-
**Nb selected* CCs endemic to time-series**	24	5	3	32
**Nb selected* CCs persistent through 4 seasons**	41	3	1	45

Table S7: Abundance and seasonality of CCs selected both Escouffier's equivalent vectors and LSP methods.

	Dataset	CC_id	mean_abundance	Year	Season
1	ASTAN	CC_unknown_12	0.05	2009	1
2	ASTAN	CC_unknown_12	0.05	2010	1
3	ASTAN	CC_unknown_12	0.05	2011	NA
4	ASTAN	CC_unknown_12	0.05	2012	NA
5	ASTAN	CC_unknown_12	0.05	2013	1
6	ASTAN	CC_unknown_12	0.05	2014	2
7	ASTAN	CC_unknown_12	0.05	2015	1
8	ASTAN	CC_unknown_12	0.05	2016	2
9	ASTAN	CC_unknown_13	0.98	2009	3
10	ASTAN	CC_unknown_13	0.98	2010	4
11	ASTAN	CC_unknown_13	0.98	2011	4
12	ASTAN	CC_unknown_13	0.98	2012	4
13	ASTAN	CC_unknown_13	0.98	2013	4
14	ASTAN	CC_unknown_13	0.98	2014	3
15	ASTAN	CC_unknown_13	0.98	2015	4
16	ASTAN	CC_unknown_13	0.98	2016	4
17	ASTAN	CC_unknown_20	0.18	2009	3
18	ASTAN	CC_unknown_20	0.18	2010	3
19	ASTAN	CC_unknown_20	0.18	2011	3
20	ASTAN	CC_unknown_20	0.18	2012	3
21	ASTAN	CC_unknown_20	0.18	2013	2
22	ASTAN	CC_unknown_20	0.18	2014	2
23	ASTAN	CC_unknown_20	0.18	2015	NA
24	ASTAN	CC_unknown_20	0.18	2016	2
25	ASTAN	CC_unknown_30	NA	2009	3
26	ASTAN	CC_unknown_30	NA	2010	1
27	ASTAN	CC_unknown_30	NA	2011	1
28	ASTAN	CC_unknown_30	NA	2012	1
29	ASTAN	CC_unknown_30	NA	2013	NA
30	ASTAN	CC_unknown_30	NA	2014	NA
31	ASTAN	CC_unknown_30	NA	2015	NA
32	ASTAN	CC_unknown_30	NA	2016	NA

33	ASTAN	CC_unknown_35	0.03	2009	1
34	ASTAN	CC_unknown_35	0.03	2010	4
35	ASTAN	CC_unknown_35	0.03	2011	2
36	ASTAN	CC_unknown_35	0.03	2012	2
37	ASTAN	CC_unknown_35	0.03	2013	1
38	ASTAN	CC_unknown_35	0.03	2014	2
39	ASTAN	CC_unknown_35	0.03	2015	2
40	ASTAN	CC_unknown_35	0.03	2016	1
41	ASTAN	CC_unknown_58	0.01	2009	3
42	ASTAN	CC_unknown_58	0.01	2010	2
43	ASTAN	CC_unknown_58	0.01	2011	2
44	ASTAN	CC_unknown_58	0.01	2012	1
45	ASTAN	CC_unknown_58	0.01	2013	1
46	ASTAN	CC_unknown_58	0.01	2014	NA
47	ASTAN	CC_unknown_58	0.01	2015	NA
48	ASTAN	CC_unknown_58	0.01	2016	1
49	ASTAN	CC_unknown_62	0.06	2009	2
50	ASTAN	CC_unknown_62	0.06	2010	3
51	ASTAN	CC_unknown_62	0.06	2011	2
52	ASTAN	CC_unknown_62	0.06	2012	4
53	ASTAN	CC_unknown_62	0.06	2013	3
54	ASTAN	CC_unknown_62	0.06	2014	3
55	ASTAN	CC_unknown_62	0.06	2015	4
56	ASTAN	CC_unknown_62	0.06	2016	2
57	ASTAN	CC_unknown_80	0.06	2009	2
58	ASTAN	CC_unknown_80	0.06	2010	2
59	ASTAN	CC_unknown_80	0.06	2011	3
60	ASTAN	CC_unknown_80	0.06	2012	3
61	ASTAN	CC_unknown_80	0.06	2013	2
62	ASTAN	CC_unknown_80	0.06	2014	2
63	ASTAN	CC_unknown_80	0.06	2015	3
64	ASTAN	CC_unknown_80	0.06	2016	4
65	ASTAN	CC_unknown_126	0.94	2009	4
66	ASTAN	CC_unknown_126	0.94	2010	4

67	ASTAN	CC_unknown_126	0.94	2011	4
68	ASTAN	CC_unknown_126	0.94	2012	4
69	ASTAN	CC_unknown_126	0.94	2013	4
70	ASTAN	CC_unknown_126	0.94	2014	4
71	ASTAN	CC_unknown_126	0.94	2015	4
72	ASTAN	CC_unknown_126	0.94	2016	4
73	ASTAN	CC_unknown_134	0.39	2009	2
74	ASTAN	CC_unknown_134	0.39	2010	4
75	ASTAN	CC_unknown_134	0.39	2011	3
76	ASTAN	CC_unknown_134	0.39	2012	2
77	ASTAN	CC_unknown_134	0.39	2013	3
78	ASTAN	CC_unknown_134	0.39	2014	4
79	ASTAN	CC_unknown_134	0.39	2015	2
80	ASTAN	CC_unknown_134	0.39	2016	2
81	ASTAN	CC_unknown_154	0.28	2009	NA
82	ASTAN	CC_unknown_154	0.28	2010	2
83	ASTAN	CC_unknown_154	0.28	2011	2
84	ASTAN	CC_unknown_154	0.28	2012	2
85	ASTAN	CC_unknown_154	0.28	2013	2
86	ASTAN	CC_unknown_154	0.28	2014	2
87	ASTAN	CC_unknown_154	0.28	2015	2
88	ASTAN	CC_unknown_154	0.28	2016	2
89	ASTAN	CC_unknown_168	NA	2009	1
90	ASTAN	CC_unknown_168	NA	2010	1
91	ASTAN	CC_unknown_168	NA	2011	NA
92	ASTAN	CC_unknown_168	NA	2012	NA
93	ASTAN	CC_unknown_168	NA	2013	NA
94	ASTAN	CC_unknown_168	NA	2014	2
95	ASTAN	CC_unknown_168	NA	2015	NA
96	ASTAN	CC_unknown_168	NA	2016	1
97	ASTAN	CC_unknown_172	0.48	2009	3
98	ASTAN	CC_unknown_172	0.48	2010	4
99	ASTAN	CC_unknown_172	0.48	2011	4
100	ASTAN	CC_unknown_172	0.48	2012	3

101	ASTAN	CC_unknown_172	0.48	2013	4
102	ASTAN	CC_unknown_172	0.48	2014	3
103	ASTAN	CC_unknown_172	0.48	2015	3
104	ASTAN	CC_unknown_172	0.48	2016	2
105	ASTAN	CC_unknown_183	0.52	2009	3
106	ASTAN	CC_unknown_183	0.52	2010	2
107	ASTAN	CC_unknown_183	0.52	2011	3
108	ASTAN	CC_unknown_183	0.52	2012	4
109	ASTAN	CC_unknown_183	0.52	2013	3
11[illegible]	ASTAN	CC_unknown_183	0.52	2014	3
111	ASTAN	CC_unknown_183	0.52	2015	3
112	ASTAN	CC_unknown_183	0.52	2016	3
113	ASTAN	CC_unknown_227	0.04	2009	1
114	ASTAN	CC_unknown_227	0.04	2010	1
115	ASTAN	CC_unknown_227	0.04	2011	NA
116	ASTAN	CC_unknown_227	0.04	2012	2
117	ASTAN	CC_unknown_227	0.04	2013	1
118	ASTAN	CC_unknown_227	0.04	2014	3
119	ASTAN	CC_unknown_227	0.04	2015	3
12[illegible]	ASTAN	CC_unknown_227	0.04	2016	4
121	ASTAN	CC_unknown_257	0.52	2009	4
122	ASTAN	CC_unknown_257	0.52	2010	4
123	ASTAN	CC_unknown_257	0.52	2011	3
124	ASTAN	CC_unknown_257	0.52	2012	4
125	ASTAN	CC_unknown_257	0.52	2013	4
126	ASTAN	CC_unknown_257	0.52	2014	3
127	ASTAN	CC_unknown_257	0.52	2015	3
128	ASTAN	CC_unknown_257	0.52	2016	3
129	ASTAN	CC_unknown_272	0.35	2009	2
13[illegible]	ASTAN	CC_unknown_272	0.35	2010	3
131	ASTAN	CC_unknown_272	0.35	2011	3
132	ASTAN	CC_unknown_272	0.35	2012	3
133	ASTAN	CC_unknown_272	0.35	2013	3
134	ASTAN	CC_unknown_272	0.35	2014	2

135	ASTAN	CC_unknown_272	0.35	2015	3
136	ASTAN	CC_unknown_272	0.35	2016	2
137	ASTAN	CC_unknown_370	0.23	2009	2
138	ASTAN	CC_unknown_370	0.23	2010	1
139	ASTAN	CC_unknown_370	0.23	2011	2
140	ASTAN	CC_unknown_370	0.23	2012	3
141	ASTAN	CC_unknown_370	0.23	2013	2
142	ASTAN	CC_unknown_370	0.23	2014	2
143	ASTAN	CC_unknown_370	0.23	2015	2
144	ASTAN	CC_unknown_370	0.23	2016	2
145	ASTAN	CC_unknown_462	NA	2009	1
146	ASTAN	CC_unknown_462	NA	2010	NA
147	ASTAN	CC_unknown_462	NA	2011	NA
148	ASTAN	CC_unknown_462	NA	2012	NA
149	ASTAN	CC_unknown_462	NA	2013	NA
150	ASTAN	CC_unknown_462	NA	2014	3
151	ASTAN	CC_unknown_462	NA	2015	2
152	ASTAN	CC_unknown_462	NA	2016	2
153	ASTAN	CC_unknown_483	0.25	2009	1
154	ASTAN	CC_unknown_483	0.25	2010	1
155	ASTAN	CC_unknown_483	0.25	2011	3
156	ASTAN	CC_unknown_483	0.25	2012	3
157	ASTAN	CC_unknown_483	0.25	2013	4
158	ASTAN	CC_unknown_483	0.25	2014	2
159	ASTAN	CC_unknown_483	0.25	2015	3
160	ASTAN	CC_unknown_483	0.25	2016	3
161	ASTAN	CC_unknown_498	0.14	2009	3
162	ASTAN	CC_unknown_498	0.14	2010	3
163	ASTAN	CC_unknown_498	0.14	2011	2
164	ASTAN	CC_unknown_498	0.14	2012	1
165	ASTAN	CC_unknown_498	0.14	2013	2
166	ASTAN	CC_unknown_498	0.14	2014	NA
167	ASTAN	CC_unknown_498	0.14	2015	2
168	ASTAN	CC_unknown_498	0.14	2016	2

169	ASTAN	CC_unknown_530	0.23	2009	4
170	ASTAN	CC_unknown_530	0.23	2010	3
171	ASTAN	CC_unknown_530	0.23	2011	1
172	ASTAN	CC_unknown_530	0.23	2012	4
173	ASTAN	CC_unknown_530	0.23	2013	3
174	ASTAN	CC_unknown_530	0.23	2014	4
175	ASTAN	CC_unknown_530	0.23	2015	4
176	ASTAN	CC_unknown_530	0.23	2016	4
177	ASTAN	CC_unknown_532	0.99	2009	3
178	ASTAN	CC_unknown_532	0.99	2010	4
179	ASTAN	CC_unknown_532	0.99	2011	4
180	ASTAN	CC_unknown_532	0.99	2012	3
181	ASTAN	CC_unknown_532	0.99	2013	4
182	ASTAN	CC_unknown_532	0.99	2014	4
183	ASTAN	CC_unknown_532	0.99	2015	4
184	ASTAN	CC_unknown_532	0.99	2016	4
185	ASTAN	CC_unknown_535	0.92	2009	3
186	ASTAN	CC_unknown_535	0.92	2010	3
187	ASTAN	CC_unknown_535	0.92	2011	3
188	ASTAN	CC_unknown_535	0.92	2012	3
189	ASTAN	CC_unknown_535	0.92	2013	2
190	ASTAN	CC_unknown_535	0.92	2014	3
191	ASTAN	CC_unknown_535	0.92	2015	3
192	ASTAN	CC_unknown_535	0.92	2016	3
193	ASTAN	CC_unknown_547	0.06	2009	2
194	ASTAN	CC_unknown_547	0.06	2010	1
195	ASTAN	CC_unknown_547	0.06	2011	2
196	ASTAN	CC_unknown_547	0.06	2012	1
197	ASTAN	CC_unknown_547	0.06	2013	1
198	ASTAN	CC_unknown_547	0.06	2014	1
199	ASTAN	CC_unknown_547	0.06	2015	2
200	ASTAN	CC_unknown_547	0.06	2016	1
201	ASTAN	CC_unknown_550	0.12	2009	2
202	ASTAN	CC_unknown_550	0.12	2010	3

203	ASTAN	CC_unknown_550	0.12	2011	2
204	ASTAN	CC_unknown_550	0.12	2012	2
205	ASTAN	CC_unknown_550	0.12	2013	3
206	ASTAN	CC_unknown_550	0.12	2014	2
207	ASTAN	CC_unknown_550	0.12	2015	3
208	ASTAN	CC_unknown_550	0.12	2016	2
209	ASTAN	CC_unknown_553	NA	2009	2
210	ASTAN	CC_unknown_553	NA	2010	1
211	ASTAN	CC_unknown_553	NA	2011	1
212	ASTAN	CC_unknown_553	NA	2012	NA
213	ASTAN	CC_unknown_553	NA	2013	NA
214	ASTAN	CC_unknown_553	NA	2014	NA
215	ASTAN	CC_unknown_553	NA	2015	NA
216	ASTAN	CC_unknown_553	NA	2016	1
217	BBMO	CC_unknown_112	0.21	2004	3
218	BBMO	CC_unknown_112	0.21	2005	3
219	BBMO	CC_unknown_112	0.21	2006	2
220	BBMO	CC_unknown_112	0.21	2007	3
221	BBMO	CC_unknown_112	0.21	2008	3
222	BBMO	CC_unknown_112	0.21	2009	3
223	BBMO	CC_unknown_112	0.21	2010	3
224	BBMO	CC_unknown_112	0.21	2011	3
225	BBMO	CC_unknown_112	0.21	2012	2
226	BBMO	CC_unknown_112	0.21	2013	2
227	BBMO	CC_unknown_126	0.35	2004	2
228	BBMO	CC_unknown_126	0.35	2005	3
229	BBMO	CC_unknown_126	0.35	2006	2
230	BBMO	CC_unknown_126	0.35	2007	4
231	BBMO	CC_unknown_126	0.35	2008	3
232	BBMO	CC_unknown_126	0.35	2009	3
233	BBMO	CC_unknown_126	0.35	2010	3
234	BBMO	CC_unknown_126	0.35	2011	4
235	BBMO	CC_unknown_126	0.35	2012	2
236	BBMO	CC_unknown_126	0.35	2013	3

237	BBMO	CC_unknown_154	0.29	2004	2
238	BBMO	CC_unknown_154	0.29	2005	3
239	BBMO	CC_unknown_154	0.29	2006	3
240	BBMO	CC_unknown_154	0.29	2007	3
241	BBMO	CC_unknown_154	0.29	2008	2
242	BBMO	CC_unknown_154	0.29	2009	2
243	BBMO	CC_unknown_154	0.29	2010	2
244	BBMO	CC_unknown_154	0.29	2011	1
245	BBMO	CC_unknown_154	0.29	2012	2
246	BBMO	CC_unknown_154	0.29	2013	2
247	BBMO	CC_unknown_213	0.02	2004	2
248	BBMO	CC_unknown_213	0.02	2005	1
249	BBMO	CC_unknown_213	0.02	2006	1
250	BBMO	CC_unknown_213	0.02	2007	2
251	BBMO	CC_unknown_213	0.02	2008	2
252	BBMO	CC_unknown_213	0.02	2009	2
253	BBMO	CC_unknown_213	0.02	2010	1
254	BBMO	CC_unknown_213	0.02	2011	1
255	BBMO	CC_unknown_213	0.02	2012	2
256	BBMO	CC_unknown_213	0.02	2013	1
257	BBMO	CC_unknown_296	0.16	2004	3
258	BBMO	CC_unknown_296	0.16	2005	2
259	BBMO	CC_unknown_296	0.16	2006	2
260	BBMO	CC_unknown_296	0.16	2007	3
261	BBMO	CC_unknown_296	0.16	2008	3
262	BBMO	CC_unknown_296	0.16	2009	2
263	BBMO	CC_unknown_296	0.16	2010	2
264	BBMO	CC_unknown_296	0.16	2011	NA
265	BBMO	CC_unknown_296	0.16	2012	2
266	BBMO	CC_unknown_296	0.16	2013	1
267	BBMO	CC_unknown_389	0.08	2004	2
268	BBMO	CC_unknown_389	0.08	2005	3
269	BBMO	CC_unknown_389	0.08	2006	2
270	BBMO	CC_unknown_389	0.08	2007	4

271	BBMO	CC_unknown_389	0.08	2008	3
272	BBMO	CC_unknown_389	0.08	2009	3
273	BBMO	CC_unknown_389	0.08	2010	2
274	BBMO	CC_unknown_389	0.08	2011	1
275	BBMO	CC_unknown_389	0.08	2012	2
276	BBMO	CC_unknown_389	0.08	2013	3
277	BBMO	CC_unknown_401	0.15	2004	2
278	BBMO	CC_unknown_401	0.15	2005	1
279	BBMO	CC_unknown_401	0.15	2006	2
280	BBMO	CC_unknown_401	0.15	2007	2
281	BBMO	CC_unknown_401	0.15	2008	3
282	BBMO	CC_unknown_401	0.15	2009	1
283	BBMO	CC_unknown_401	0.15	2010	1
284	BBMO	CC_unknown_401	0.15	2011	1
285	BBMO	CC_unknown_401	0.15	2012	2
286	BBMO	CC_unknown_401	0.15	2013	1
287	SOLA	CC_unknown_126	0.77	2009	3
288	SOLA	CC_unknown_126	0.77	2010	3
289	SOLA	CC_unknown_126	0.77	2011	3
290	SOLA	CC_unknown_126	0.77	2012	3
291	SOLA	CC_unknown_126	0.77	2013	3
292	SOLA	CC_unknown_126	0.77	2014	3
293	SOLA	CC_unknown_143	0.42	2009	2
294	SOLA	CC_unknown_143	0.42	2010	1
295	SOLA	CC_unknown_143	0.42	2011	2
296	SOLA	CC_unknown_143	0.42	2012	2
297	SOLA	CC_unknown_143	0.42	2013	3
298	SOLA	CC_unknown_143	0.42	2014	2
299	SOLA	CC_unknown_149	0.41	2009	3
300	SOLA	CC_unknown_149	0.41	2010	3
301	SOLA	CC_unknown_149	0.41	2011	4
302	SOLA	CC_unknown_149	0.41	2012	2
303	SOLA	CC_unknown_149	0.41	2013	2
304	SOLA	CC_unknown_149	0.41	2014	2

305	SOLA	CC_unknown_183	0.48	2009	3
306	SOLA	CC_unknown_183	0.48	2010	2
307	SOLA	CC_unknown_183	0.48	2011	1
308	SOLA	CC_unknown_183	0.48	2012	3
309	SOLA	CC_unknown_183	0.48	2013	3
310	SOLA	CC_unknown_183	0.48	2014	2
311	SOLA	CC_unknown_425	0.21	2009	2
312	SOLA	CC_unknown_425	0.21	2010	3
313	SOLA	CC_unknown_425	0.21	2011	2
314	SOLA	CC_unknown_425	0.21	2012	3
315	SOLA	CC_unknown_425	0.21	2013	2
316	SOLA	CC_unknown_425	0.21	2014	2
